# Extra Supplement.—The Nursing Mirror

**Published:** 1888-12-29

**Authors:** 


### The Nursing Mirror.

All Contributions for this Supplement should be addressed "The Nursing Mirror," care of The Editor, 38, Old Jewry, E.C.

### En Passant.

RICHES AND POVERTY.—It is said that while Lady Herbert, of Lea, was in New York she was surprised to meet in the garb of a Little Sister of the Poor, a young lady who a few years ago was one of the wealthiest belles of the London season. The young lady did well to go to New York when relinquishing her high position. Some of our dames of fashion who, in search of a new sensation, go and potter about the wards of St. George's in a cap and apron would do better if they did the thing thoroughly or else left it entirely alone. Dilletantism has no place in a profession like nursing.

SHORT ITEMS.—Mrs. Clifton Dicconson, of Wrightington Hall, has been asked to present the certificates to the nurses trained this year at the Wigan Infirmary. —The guardians of Lambeth permitted the infirmary nurses to hold a little levée on Christmas Day.—The report just issued of the Dublin Orthopædic Hospital speaks most kindly of the late long illness of the lady superintendent.—At the annual meeting of the committee of the Midland Counties Home for Incurables, General Radcliffe said they had a most excellent and conscientious matron in the daughter of Lord Godolphin Osborne, who did her work thoroughly well.

LECTURES TO INFIRMARY NURSES.—Boards of guardians are indeed becoming enlightened when they grant £10 towards teaching their nurses. The Medical Superintendent of Fulham Infirmary having offered to lecture to the nurses and assistant nurses on the lines laid down by the St. John's Ambulance Association, the guardians sanctioned the proposal and granted the above sum for the purchase of necessary books, diagrams, and a skeleton. The importance of this move cannot be over-rated, for the Sairey Gamps who yet linger in our ranks are mostly to be found in workhouse infirmaries, and if knowledge be demanded of them, they will soon disappear even from those corners.

SOIREE AT "THE LONDON."—On December 20th a very pleasant conversazione was given by Mr. Carr Gomm to the nursing staff of the London Hospital. Besides a large gathering of nurses, there were present several of the medical staff, past and present, four members of the committee, some of the neighbouring clergy, and others. The gathering was held in the large handsome library of the Medical College, and the band of the Royal West Surrey Volunteers (Queen's) was in attendance. During the day rumours had circulated that Mr. Stuart Cumberland was ill, and would not be able to give the promised thought-reading, but, contrary to expectations, Mr. Cumberland managed to put in an appearance, and though looking very ill, gave two excellent illustrations of his extraordinary powers. The first was a supposed murder scene, the second a supposed injury, which had to be bandaged. One of the house-surgeons acted as medium in the second illustration, and truly seemed astonished at the dashing rapidity with which Mr. Cumberland carried out his thoughts. During the evening four of the students sang some glees, and a conjuror gave an amusing entertainment, during which he victimised two of the guests to a laughable extent. Refreshments were served in the dining-hall, and altogether the evening proved a great success, and was a welcome break in the monotonous round of duty which makes a nurse's life, and also a seasonable taste of Christmas festivities for those who know how to relish them.

NURSES AT ALEXANDRIA.—The *Times* for December 19th contained an account of the Victoria House and Nurses' Home, from an Alexandria correspondent. Two English-trained nurses live in the home and undertake private nursing. They have been actively employed during little less than half the time, and their services have been much appreciated. The Khedive has lately presented the committee with a suitable site on which it is soon hoped a permanent home will be built: for this purpose subscriptions are being received by the British Consul and others. Three thousand pounds is required for building purposes, and it is then believed the home would prove self-supporting.

HOSPITAL EXTRAVAGANCE.—Amongst the other points touched on in Mr. Michelli's paper, which is being so much discussed, was that of the extras granted to patients at the request of the sister. The *Pall Mall Gazette* put the matter in an unnecessarily unpleasant form: "The friendship which sometimes springs up between clever and amiable nursing 'Sisters' and the members of the resident medical staff is responsible for one of the milder forms of extravagance. These young ladies often think 'extras' of various kinds very desirable for their patients; and on their own authority will ask for such extras. The resident medical officer, young and susceptible, cannot refuse a request made by a pleasant-faced woman, also young; and so the 'extras' are ordered by the doctor without regard either to the special necessities of the patient or the funds of the hospital." Of course a sister is responsible for the amount of petty waste which goes on in her ward, but she seldom interferes in the orders of the medical staff. Hospitals are not hot-beds of luxury, and if a sister does sometimes take it upon herself to request an extra egg for some patient, surely the physician will not give it, if it is absolutely unnecessary. Expenditure may be wrongly kept down by cutting off patients' extra food, instead of by reducing the management expenses.

PHILANTHROPIC SWEATING.—Under this heading the *Daily News* has printed a letter from some nurses, grumbling at their work and diet. They formulate their complaint as follows: "Some people think a nurse's life is all sunshine, but it is not. First of all they have over them people who do not know how to treat them, take every opportunity of bullying them and making them perfectly miserable; as for having any rest, never think they want any. From early morning till late at night they must keep on at work. As for food, they are told anything is good enough for them, and they find it has to be unless bought by themselves, which is rather hard lines, as a hospital nurse is not very well paid." That a nurse's life is a hard one we are all bound to acknowledge, but it is also a beautiful one, and full of varying interest. It has many compensations for the physical and mental strain it lays on its followers, and to bring a vague anonymous charge against the authorities at large, can lead to no good. How many a matron is striving her very best to make the lives of her nurses pleasant and comfortable; and even committees are considering how wages can be increased, pensions procured, and better food provided. Reforms cannot be completed in a day, and looking back only a very few years, we ought to be grateful to the authorities in the nursing world for the changes they have wrought. If the nurses in question have any definite charges to make, and will write to us in confidence, we will make inquiries, and ascertain whence the wrongs spring of which thay so bitterly complain.

### Letters from a Probationer.

### No. VI.—Matron at Home.

Dear——, Doubtless you must have wondered that in all my letters there has been no mention of the matron. The fact is, I was terribly afraid of matron at first, she is so tall and stately, and has such quick eyes for detecting aught amiss. To begin with a personal description such as is interesting to a woman I may remark that matron is well under forty, and that on my first interview I searched in vain for grey hairs to reverence. Her eyes are very dark and bright, and she wears a most imposing and becoming cap with long tails; her voice is very clear but low, and she moves quickly but gracefully. All these are mere details though, it is when you come to inward graces that the character of matron shines forth in its glory. She is justice itself, yet she knows mercy at times, and her memory is a perfect marvel. All these months I thought myself quite unknown to her personally, till this very evening I summoned up courage to go to her "at home." Every Wednesday evening matron is at home in her own pretty little parlour to any nurse who chooses to go and see her. She sits behind a cosy tea-table and pours out tea herself, and generally some favoured pupil is allowed to sit by her side and dispense the milk and sugar. I was astonished to be greeted with an extended hand and an—"At last, my dear!" I thought you were never coming to see me."

"But matron, you must always have so many visitors. I didn't like to intrude."

"I never get too many of my nurses at a time, the ward duties prevent that. I suppose you have left Nurse Wallace in charge?"

"Yes, matron," I replied wonderingly, for moving about from ward to ward as probationers do, it was extraordinary to me that matron should remember where I now am, and the name of my fellow nurse. I drank my tea and listened to the conversation which matron carefully led away from hospital cares and keep on cheerful topics. There was plenty of laughter, and the group round the fire would have made a pretty picture. One nurse was sitting on a stool at Matron's feet and nursing the cat; another was wandering round the room looking at the photographs and nick-nacks which decorated every corner. The matron who sweeps through the wards daily with keen glance, detecting untidiness, and the matron who sits at the desk in the office, and interviews would-be pros in such a business-like manner, vanished from my memory, and instead of either I saw what seemed a group gathered together round their familiar friend.

I have been asking Nurse Wallace since I have come back to the ward, if other matrons are all like ours, and she answers emphatically, "No?" that nowhere else is there such kindness and consideration blended with thorough discipline and training as here. The comfort of the nurses is considered in moderation, their character and work is considered at every turn. You know I found the strictness irksome at first, and the lack of luxury trying; now I can see the matter from a better point of view, it becomes evident to me that such a large band of women are best managed by hard and fast rules, and that plain living is good for our health at least. I have only one grumble left: I *do* want an extra hour's sleep every night. Nurse Wallace says that in other hospitals there may be soirèes for the nurses at Christmas, and the matron may get up an entertainment for them occasionally, but that the constant friendly interest and helpful counsel matron is always ready to give to us here, is to be found in very few hospitals. There! I couldn't go to bed to-night till I had told you this, so struck was I this evening, with my first visit to matron "at home."

### Notes from Australia.

(From Our Own Correspondent.)

Melbourne, October 19th.

The Benevolent Asylum, Melbourne, is situated in a commanding position in one of the suburbs, Hotham. The front of the building looks down a broad street towards Melbourne, and from the upper windows are good views of Williamston, the shipping in the bay, and the country around. As one nears the garden gate there are numbers of old men to be seen, some with sticks, some with crutches, some without. They all look more or less interested to see a stranger coming, and the one who opened the gate evidently had an idea of his own and the asylum's importance. A home which comfortably shelters 656 of the aged and infirm may be rightly considered of importance. The Benevolent Asylum was founded June 24th, 1850. In the chief entrance hall there is a slab of white marble enumera ting the gifts of various friends. During the past year (1887) bequests have been made amounting to £4,609 2s. 11d. for the endowment fund. The present superintendent takes great interest in his work, and does it well. He has the look of an old English gentleman, with a history. Several of the patients are quite invalids; some are able to knit or sew. One very stout woman had learnt to knit since she came to the asylum. "That young woman in the corner taught me." The young woman was not less than 50! In another ward we were formally introduced to Mrs. Brandon, who is 100 years old; this has been her home since 1866. She looks well enough for it to be so for 20 years more; She sews and reads, and has pleasure in criticising the fashions of the present day in the matter of dress. One old lady was reading "Madcap Violet." "It's such an interesting book," said she. On my saying "Yes, but the end is very sad," she answered "Ah, I havn't got to that yet," in a tone that meant I only look at the shadows of life when I'm obliged to. One could not help feeling very sorry for the weary hours that some of the inmates have to suffer; and also glad that some of the old Colonists are good in remembering their less fortunate brothers and sisters. Among these is Mr. Westgarth, who is now just returning to England after a visit of some months in Australia. Mr. Westgarth was the first president of the Melbourne Chamber of Commerce, and the prime mover in the establishment of a chamber of commerce in London.

It certainly does seem a pity something cannot be more quickly done to lessen typhoid and diphtheria. With such a splendid climate as Victoria, we ought not to be so afflicted with these terrible diseases. Within a very short time there have been two cases of only children of wealthy parents carried off by diphtheria to my own knowledge. We are all wild with the hope that the cure is now being discovered in the Eucalyptus; and if so, the folly of meddling with Nature will once more be made plain, for diphtheria will be proved to be the punishment for the ruthless destruction of forests of Eucalyptus which formerly abounded in these regions. It is to be hoped that Lady Samuels efforts to help nursing in Sydney may be accepted, and may be extended to Victoria and South Australia. In Melbourne, at least, though efforts are being made for the instruction of nurses, there is much jealousy to contend with, and much ignorance about nursing. Also, there is what is greatly to be regretted, a tendency among young people in good positions to leave the sick and elderly people to the care of servants, while they go to the endless amusements of Melbourne. If this tendency increases, there must be more demand than even now for good nurses. There is a noble band of workers, but these be few, and what are they among so many?

The Women's Hospital is so full that at the weekly meeting, October 14th, 1888, it was feared that unless further provision was made many applicants would have to be turned away.

At the Melbourne Hospital there appears to be every prospect of a very gratifying success attending the efforts of Mr. Derbin Willder and the committee to reduce the debt on the hospital. The anticipation that a number of the citizens who have shared largely in the exceptional prosperity of this year might be induced to contribute sums of £100 each towards relieving the institution from its serious encumbrance is being realised to an encouraging degree. Within two days twenty-two contributions of £100 each have been announced.

At the monthly meeting of the British Medical Association, Sept. 26th, a paper on "Our Lunatic Asylums" was read by Dr. W. H. Barker, superintendent of Kew Asylum, dealing with the merits of the cottage and barrack system. Dr. Barker is in favour of the last, which is common in England, but the Victorian Government favours the first.

The Central Board of Health has hard work to get all its wants fulfilled. People will not be convinced of the importance of getting rubbish thoroughly cleared away. and other sanitary matters are ignored when they should be acted on.

The Australian Health Society is doing very good work in the distribution of pamphlets on "Pure Air and Ventilation," "Rules for the Management of Infants," etc., and in having lectures in the Young Men's Christian Association rooms.

### The National Pension Fund for Nurses.

Our attention has only just been called to the annotation which appeared in the *Medical Press and Circular* on November 28th. We think the editors can hardly be aware that it was reported to the last meeting of the Council that already 575 nurses had applied for policies in addition to those from one institution having 105 nurses which had decided to affiliate itself with the Fund to pay in a lump sum of £500, an annual contribution of nearly £200, and £2 per head for all the younger nurses. This brings the number of proposals up to 680, leaving only 320 to complete the one thousand which it is desired to secure before the end of January, 1890. The invested funds already amount to upwards of £30,000. Our readers may remember that it was only decided to accept proposals in July last, so that the 680 have come in in less than six months, a remarkable testimony to the thrifty spirit which animates the better class of nurses, knowing as we do that the most careful calculations go to prove that there cannot be more than 10,000 nurses at the outside who are in a position to join the Pension Fund. The originators of this Fund had therefore a high estimate of the character of nurses as a body, or they would never have ventured to require that 1,000 nurses, that is, one in every ten able to do so, should consent to pay into the Fund before the £20,000 should become applicable to their benefit. For these reasons the prospects of the National Pension Fund are at present not only not gloomy, but inspiriting and satisfactory to the fullest extent. We desire, however, to take this opportunity of thanking our contemporary for the kindly support it has extended to this movement in the interests of nurses, and to welcome its kindly criticism in the interests of the Fund. The original prospectus was put forward in a certain form, with the object of impressing upon nurses the business-like aspect in which it was desired that they should regard this Fund. Several so-called friends of nurses have desired that they should be degraded to the level of mere recipients of doles from the wealthy, by insisting that nurses were unable to help themselves at all, and that therefore they must be provided with a huge charity fund, upon which every nurse could fall back when she thought she required pecuniary assistance. The promoters of the Pension Fund are fully alive to the fact that the earnings of nurses are relatively small, but they recognise that there is a margin for saving out of their earnings which can properly and beneficially be invested through a central fund for their benefit. It is felt, however, that the best paid nurse who is over 30 will probably find herself unable to contribute enough to purchase an annuity or pension for herself at an age when she may justly retire from work. Hence the contribution of £25,000 to the Fund by a few merchant princes, and the determination to augment this endowment fund, by the free gifts of a grateful public, to an extent adequate to yield every nurse who joins the Pension Fund a retiring allowance which will enable her to end her days in independence and comfort.

The merchant princes, and those who are acting with them in this matter, have desired to keep the benevolent aspect of the Pension Fund in the background until it could be ascertained whether the great body of nurses possessed sufficient thrift to warrant the establishment of a national fund for their benefit. It is this feeling which has prevented the promoters from canvassing the nurses or visiting the hospitals up to this time, and the high opinion they hold of nurses is abundantly justified by the knowledge that, despite the reticience to which the *Medical Press* calls attention, new proposals are being spontaneously taken up at the rate of at least sixty per month. This is a substantial evidence of the thrift to be found amongst nurses, and there is therefore no longer any reason to refrain from adopting the suggestion to appoint two or more gentlemen, who are widely interested and posted up in the scheme, to visit each hospital from which proposals have not been received, and to demonstrate to the nurses the advantages which the Fund affords them. This plan will shortly be put into execution, and if any of our readers who are attached to institutions, and who are interested in nurses will ascertain when it would be convenient and agreeable to receive a representative of the Council, and if they will kindly communicate the fact to Mr. Edward Clifford, 8, King Street, E.C. (Honorary Manager), every effort will be made to meet the wishes and convenience of the authorities and nurses throughout the country.

It is suggested, as there are usually at least one hospital and one nursing institution in every town of any size, it would save time if arrangements could be made to assemble the nursing staff of all the institutions in each district in some central room at one time, to confer with the deputation. By this means any doubts or difficulties which may have presented themselves to the minds of those who have heard of the Fund and wish to know more about it would soon be cleared up. The great thing to bear in mind is that a nurse over 30 is not expected (because it is known, as a rule, to be impossible) to contribute a sum to the Pension Fund sufficient to produce an adequate pension at a certain age, according to the tables. On the contrary, all that nurses are expected to do is to join the fund by contributing something, that is, the utmost they can spare, from their earnings, and then we have a distinct reason for hoping that the Fund will be able to supplement their contributions to a sufficient extent to provide them with a pension when incapacitated for work and for the rest of their lives. It is particularly wished that lady superintendents and matrons will take up this suggestion, and arrange a meeting at the end of January or in February.

### Everybody's Opinion.

*Correspondence on all subjects is invited. No communications can be entertained if the name and address of the correspondent is not given' or unless one side of the paper only be written on.*]

### REST AND EXERCISE.

A nurse belonging to one of the Provincial Nursing Institutions desires a few words on the important subjects of rest and exercise. At the present time she is nursing a chronic case. The rules of the institution say that "when the nurse is required for several consecutive nights, she shall have seven hours' rest in the day, out of the sick-room." No time is specified in addition to this for exercise, and the nurse consequently suffers. She comes on duty at 9 p.m., and is not relieved until 2 p.m. the next day, and oftener it is 2.15 p.m. Then, if nurse chooses to take a short walk, it must come out of her seven hours, and she does not often get to rest until after 3 p.m. She has to appear, having had supper and ready for the night, at 9 p.m. To do this, she is obliged to rise at 8 p.m., thus leaving her five hours for rest. She must not complain at her case, and if she complains at the institution she is soon told that if she does not like the rules she may leave them. Is it not a great pity that after a nurse has been on duty from 9 p.m. till 12 noon—15 hours—she is not allowed time for a blow of fresh air, without taking the time in which she is supposed and ought to sleep? Some institutions specify that the nurse be allowed two hours daily outdoor exercise, and that as often as the state of the patient may allow, she may attend Divine worship. If all institutions would fix this rule, it would save the nurses a great deal of trouble and loss of health. I broke down a short time ago, and what happened? Why, I was sent for a holiday at my own expense!

### Lectures.

At the Royal Institution, Albemarle Street, at three p.m., George John Romanes, Esq., M.A., LL.D., F.R.S., M.R.I., Fullerian Professor of Physiology, R.I. Twelve lectures, constituting the second part of a course on "Before and After Darwin (the Evidences of Organic Evolution and the Theory of Natural Selection)," on Tuesdays, January 22nd to April 9th. One guinea the course.

At the Sanitary Institute, Margaret Street, five p.m., Dr. G. V. Poore on "London, Ancient and Modern, from a Sanitary Point of View," on January 24th. Admission 6d. Professor F. Jeffery Bell, on "The Worm Parasites of Human Food," on January 31st. Admission 6d.

At B.N.A. Rooms, 11, Chandos Street, at eight p.m., R. Brudenell Carter, Esq., F.R.C.S., on "Some Scientific Aspects of Truthfulness," on January 18th. Admission to non-members, 1s.

### Appointments.

[It is requested that successful candidates will send a copy of their applications and testimonials, with date of election, to The Editor, The Lodge, Porchester Square, W.]

Nurses' Home, Newcastle.—Miss E. Emery has been appointed matron of the Nurses Home and Training School, Newcastle-on-Tyne. Miss Emery has been engaged in nursing for many years, and possesses numerous excellent testimonials.

Armagh Infirmary.—Miss Mary Taylor, trained at Sir Patrick Drew's Hospital, Dublin, has been appointed lady superintendent of nurses and matrons of this infirmary.

### The Nurses' Bookshelf.

[Books for Review should be sent to The Editor, The Lodge, Porchester Square, W.]

### Laying Out the Dead.*

The subject of this small pamphlet is one from which a woman may well shrink, but which has to be faced by every nurse. And when a thing has to be done, it may as well be done properly and thoroughly: there is a right and a wrong way of doing everything; those who wish to learn the right way of performing the last offices for the dead, cannot do better than read and learn from Miss Pierrepont's pamphlet. There is no unnecessary writing in the four pages which lie before us, yet there is no single direction or word of help left unsaid. Miss Pierrepont goes straight to the matter in hand: "As soon as the spirit has left the body, try with love and gentleness to persuade the loved ones to leave the body to your care and have the room cleared, leaving only two besides yourself. Close the eyes at once by gentle pressure, and keep your thumb and first finger on them until you find they will not re-open, then take a clean white handkerchief, pass it under the jaws, and tie it tightly on the top of the head; take a second handkerchief and tie the feet together above the ankle." This last direction is one commonly omitted, and is yet necessary. Directions for clearing and ventilating the room, and for disinfecting the bedding are then given; and then an excellent method of washing the body with decency and care is fully described. All the little touches which do so much to take away from the ghastliness and horror of death are dwelt on by Miss Pierrepont, and the nurse is directed herself to see that the grate is cleared, all medicine bottles, etc., put away, and clean water and towels left for the undertaker. In conclusion, the nurse is advised never to thwart in any way the wishes of friends and relatives, and to avoid talking about the dead.

This pamphlet was written by request for the Nursing Guild of St. Veronica, but will be welcomed by many nurses who are not members of the guild.

### Vacancies.

The following vacancies are announced:—

**Matrons.**

Bolton Infirmary and Dispensary; £75

Jersey General Dispensary; £25.

Ladies Charity and Lying-in Hospital, Brownlow Hill. Liverpool

Southend Victoria Hospital; £30

Ulverston and District Cottage Hospital; £35.

(assistant).

**Sister.**

Royal Hospital for Children and Women, Waterlow Bridge Road; £30

**Nurses.**

Bedford Nursing Institute; two: £24.

Nursing Institution, 96, 97, 98, Wimpole Street, London, W., Mr. Wilson has several vacancies for thoroughly-trained Nurses.

Croydon General Hospital; £22.

Victoria Home for Nurses, Bournemouth; Several.

Southport Nurses Home; several.

Christ's Hospital, Hertford; £20.

Home for Epileptics, Maghull; £20.

Hartlepools Hospital, Night; £25.

Nurses' Home, Beckenham.

Cheltenham Nursing Institution.

Swansea Nursing Institution.

Windsor Royal Infirmary; £20.

General Nursing Institute, 5, Henrietta Street, Covent Garden; Several.

Home Hospital, Eastbourne.

Royal Hospital for Children and Women.

**District Nurses.**

Newlands, Ryde.
Cotswold House, Gloucester.

**Midwives.**

Manchester Maternity Hospital; two.

Newlands, Ryde.

Wrexham, Wales.

**Probationer.**

Royal Hospital for Children and Women.

### Notes and Queries.

To Correspondents.—1. Questions may be written on post-cards. 2. Advertisements in disguise are inadmissible. 3. In answering a query please quote the number. 4. If a private answer is desired, a stamped addressed envelope must be forwarded.

**Queries.**

(93) Information wanted as to how to pack a mental patient.

(94) I have to take my holiday early next year: what is the best place to go to? It must be economical, and the fare there and back not too great.—*Nurse James.*

**Answer.**

(91) *Male Nurses.*—The Hamilton Association, 22, South Audley Street, trains male nurses.

* "Laying Out the Dead." By S. A. C. Pierrepont. (W. Leicester, Worcester.) Price 3d.

